# The Effect of Creosote on the Virulence of the Tuberculii Bacillus

**Published:** 1894-12

**Authors:** 


THE EFFECT OF CREOSOTE ON THE VIRULENCE OF THE TUBERCLE BACILLUS. (*British Medical Journal*, September 22, 1894.) By W. Kington Fyffe, M. B. (Cantab.)—In this paper the writer gives us the results he obtained from an investigation undertaken to decide whether the value of creosote, as a remedy in phthisis, was due to any restraining action exercised over the growth of the bacillus, or simply to its power of improving digestion, and so aiding assimilation. At Victoria Park Hospital, creosote is used in one of three ways—(1) as an inhalation; (2) by the mouth; (3) by means of a creosote chamber. The latter method consists in placing a patient in a small room, in which creosote is heated till the air is saturated with the vapor. The method adopted by the writer was that recommended by Professor Delapine—namely, that of injecting tuberculous sputum into the leg of a guinea-pig, and noting the time it lived, and the extent of lesion at the time of death. The conclusions he arrived at are summed up as follows: (1) In those patients who were taking creosote simply as an inhalation, no effect on the virulence of the bacilli was noted. (2) In those to whom the creosote was administered by the mouth, in doses ranging from two to twelve minims, three times a day, a slight diminution in virulence was noted when the dose was small, while when larger amounts were administered, the diminution was well marked. (3) In those to whom the creosote was administered by the third method, the number of cases examined was only three, and therefore an absolute dictum could hardly be given, yet the animals lived longer after the injection than in any other instance, and died in the end from cellulitis, set up by the injections.—*Ex.*

